# Predictors of ventricular tachyarrhythmia in patients with a wearable cardioverter defibrillator: an international multicenter registry

**DOI:** 10.1007/s10840-024-01869-w

**Published:** 2024-07-10

**Authors:** Fabienne Kreimer, Katharina Koepsel, Michael Gotzmann, Boldizsar Kovacs, Tobias C. Dreher, Christian Blockhaus, Norbert Klein, Thomas Kuntz, Dong-In Shin, Hendrik Lapp, Stephanie Rosenkaimer, Mohammad Abumayyaleh, Nazha Hamdani, Ardan Muammer Saguner, Julia W. Erath, Firat Duru, Thomas Beiert, Fabian Schiedat, Christian Weth, Florian Custodis, Ibrahim Akin, Andreas Mügge, Assem Aweimer, Ibrahim El-Battrawy

**Affiliations:** 1 Department of Cardiology and Rhythmology, University Hospital St. Josef-Hospital Bochum, Ruhr University Bochum, Gudrunstraße 56, 44791 Bochum, Germany; 2 Department of Cardiology, University Hospital Münster, Münster, Germany; 3 Department of Cardiology and Angiology, Bergmannsheil University Hospital, Ruhr University of Bochum, Bochum, Germany; 4 Department of Cardiology University Heart Center, University Hospital Zurich, Zurich, Switzerland; 5 Center for Translational and Experimental Cardiology (CTEC), Department of Cardiology, Zurich University Hospital, University of Zurich, 8952 Schlieren, Switzerland; 6 Department of Cardiology, Angiology, Haemostaseology and Medical Intensive Care University Medical Center Mannheim, Medical Faculty Mannheim, Heidelberg University, Mannheim, Germany; 7 Department of Cardiology Heart Centre Niederrhein, Helios Clinic Krefeld, Krefeld, Germany; 8 Faculty of Health, School of Medicine, University Witten/Herdecke, Witten, Germany; 9 Department of Cardiology, Angiology and Internal Intensive-Care Medicine, Klinikum St. Georg gGmbH Leipzig, Leipzig, Germany; 10 Department of Internal Medicine II, Heart Center Bonn, University Hospital Bonn, Bonn, Germany; 11 German Center for Cardiovascular Research (DZHK), Partner Site Heidelberg-Mannheim, Mannheim, Germany; 12 Institute of Physiology, Department of Cellular and Translational Physiology and Institut Für Forschung Und Lehre (IFL), Molecular and Experimental Cardiology, Ruhr-University Bochum, Bochum, Germany; 13 Department of Cardiology, Frankfurt University Hospital Goethe University, Frankfurt Am Main, Germany; 14 Department of Cardiology, Marienhospital Gelsenkirchen, Academic Hospital of the Ruhr University Bochum, Bochum, Germany; 15 Department of Internal Medicine II, Klinikum Saarbruecken, Saarbruecken, Germany

**Keywords:** Wearable cardioverter defibrillator, Predictors, Ventricular tachycardia, Ventricular fibrillation, Sudden cardiac death

## Abstract

**Background and aims:**

Wearable cardioverter defibrillator (WCD) can protect patients from sudden cardiac death due to ventricular tachyarrhythmias and serve as a bridge to decision of definite defibrillator implantation. The aim of this analysis from an international, multicenter WCD registry was to identify predictors of sustained ventricular tachycardia (VT) and/or ventricular fibrillation (VF) in this population.

**Methods:**

One thousand six hundred seventy-five patients with WCD were included in a multicenter registry from 9 European centers, with a median follow-up of 440 days (IQR 120–893). The primary study end point was the occurrence of sustained VT/VF.

**Results:**

Sustained VT was detected by WCD in 5.4% and VF in 0.9% of all patients. Of the 30.3% of patients receiving ICD implantation during follow-up, sustained VT was recorded in 9.3% and VF in 2.6%. Non-ischemic cardiomyopathy (HR 0.5, *p* < 0.001), and medication with angiotensin-converting enzyme inhibitors (HR 0.7, *p* = 0.027) and aldosterone antagonists (HR 0.7, *p* = 0.005) were associated with a significantly lower risk of VT/VF.

**Conclusions:**

Patients who received WCD due to a transient increased risk of sudden cardiac death have a comparatively lower risk of VT/VF in the presence of non-ischemic cardiomyopathy. Of note, optimal medical treatment for heart failure not only results in an improvement in left ventricular ejection fraction but also in a reduction in the risk for VT/VF.

**Graphical Abstract:**

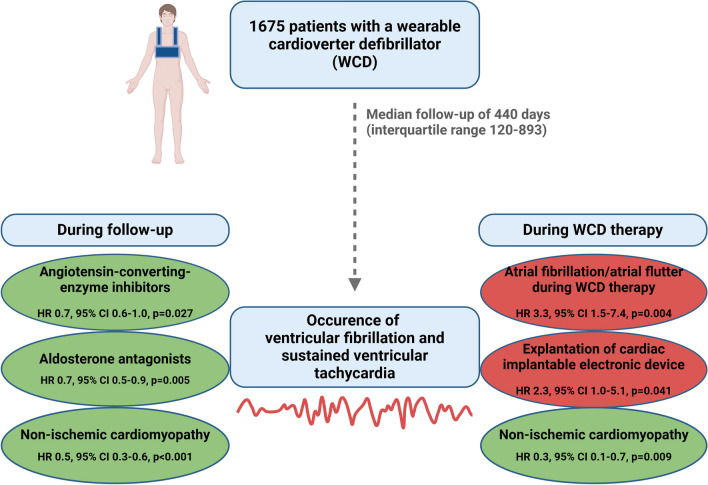

**Supplementary Information:**

The online version contains supplementary material available at 10.1007/s10840-024-01869-w.

## Introduction

Patients with a reduced left ventricular ejection fraction (LVEF) ≤ 35% are at increased risk of sudden cardiac death (SCD) [1, 2]. Some patients may experience an improvement in LVEF after optimal medical treatment (OMT), leading current guidelines to recommend a waiting period of 6 to 12 weeks (depending on the underlying cause) after the initial diagnosis of severely reduced LVEF before considering definite implantable cardioverter defibrillator (ICD) implantation [3]. During this treatment period, patients remain at a heightened risk of SCD [2]. However, despite OMT, some residual SCD risk may persist [1, 3]. Data have shown that the LVEF is not the only predictor for SCD or mortality, but in addition, the disease’s etiology is playing an important role [4].

While ICDs are known to enhance survival and reduce mortality due to ventricular tachyarrhythmias, some patients may not meet the criteria for ICD implantation [2]. Studies like IRIS and DINAMIT demonstrated that early ICD implantation after acute coronary syndrome does not significantly improve survival rates [5, 6]. Moreover, although ICD implantation is possible in young patients, it comes with various device-related complications, including inappropriate shocks, infections, lead failure, and lead dysfunction, which tend to increase over time. These complications could be avoided by limiting unnecessary ICD implantations [7–9].

Wearable cardioverter defibrillators (WCDs) have potential indications for both secondary and primary prevention in patients with ischemic (ICM) or non-ischemic cardiomyopathies (NICM), as well as for those who have had infected ICDs removed or who have experienced myocarditis, peripartum cardiomyopathy, or other cardiomyopathies [8–12]. WCD could serve as a safeguard for these patients against cardiac arrest and as a bridge to a decision regarding ICD implantation [13]. Several reports suggested that WCDs could potentially obviate the need for permanent ICD implantation in a significant portion of patients [14].

However, data from the VEST trial suggest that WCDs do not significantly impact arrhythmic death as a primary endpoint after up to 90 days of use in patients with myocardial infarction and moderate to severe left ventricular dysfunction compared to controls. While there was a significant reduction in all-cause mortality (a secondary endpoint) and a low rate of inappropriate shocks in the WCD group, the published data do not support the systematic unselected use of WCDs in this patient population [15].

Therefore, the question arises which patients are particularly at risk of developing sustained ventricular arrhythmias and arrhythmic death and would benefit from an ICD. The aim of this analysis of a multicenter, international WCD registry of 1675 patients was to identify predictors of sustained ventricular tachyarrhythmia.

## Methods

### Study design

Between April 2012 and December 2022, we included 1675 patients who received a WCD (ZOLL Life Vest™ system) in 9 centers in Germany and Switzerland (Bergmannsheil University Hospital, University Hospital Zurich, University Hospital Mannheim, Helios Clinic Krefeld, University Hospital St. Josef‐Hospital Bochum, Klinikum St. Georg Leipzig, University Hospital Bonn, Frankfurt University Hospital, Klinikum Saarbrücken). The primary study end point was the occurrence of sustained ventricular tachycardia or ventricular fibrillation (VT/VF), either detected by WCD or occurring during subsequent follow-up. We differentiated between sustained VT/VF, which occurred exclusively during the WCD wearing period, and all sustained VT/VF episodes, i.e., both during the WCD wearing period and during follow-up in patients with ICD implantation.

This study was conducted in accordance with the Declaration of Helsinki on Human Investigations, and the study protocol was approved by the ethics committees of all participating centers.

### The wearable-cardioverter-defibrillator (WCD)

The WCD ZOLL Life Vest™ system and programmed data have recently been described in detail [10]. During programming, several issues were considered, including underlying cardiac disease and electrocardiographic patterns. In general, in older patients, the VT zone was programmed with a heart rate of 150 to 190 beats per minute and a VT response time of 60 s, whereas in younger patients, a VT zone was programmed with a heart rate of 180 to 190 beats per minute and a VT response time of 60 s as well. The VF zone was similarly programmed in older and younger patients with a heart rate of 200–220 beats per minute and a response time of 25 s. The maximum energy of the first shock was 150 J, and episodes were recorded with a minimum delay of 3 min. Episodes were reviewed and classified by physicians. Episodes were divided into two groups: sustained VT (duration 30 s or longer) or VF with WCD shock therapy and non-sustained VT (duration less than 30 s) without WCD shock. Inappropriate WCD therapy was identified as non-ventricular tachyarrhythmias or non-ventricular fibrillation episodes treated with an inappropriate WCD shock.

### Baseline and follow-up data collection

Baseline characteristics of patients were assessed at each center. All clinical data were retrospectively collected locally and all WCD data were retrieved from the ZOLL Life Vest Network™. The WCD was prescribed by physicians according to current guidelines and with assessment of risk for sudden cardiac death, e.g., LVEF ≤ 35%. Regardless of the underlying heart disease, the use of a WCD was recommended for 3 months. WCD wearing time and WCD shocks during WCD use were documented. Compliance was defined as wearing more than 20 h per day.

For follow-up data, treating physicians and/or patients were contacted. When feasible, index LVEF, a follow-up LVEF at 3 months (short-term), and at 6 to 12 months (long-term) were assessed and calculated by the biplane Simpson method using echocardiography and/or cardiac magnetic resonance imaging (MRI). Improvement was assumed if an increase in LVEF to 36% or more was observed during follow-up. Each center and physician decided whether prolongation of WCD use was necessary or useful based on different considerations, e.g., in some cases of relevant improvement in LVEF but still LVEF ≤ 35%. OMT was achieved with commonly recommended heart failure medications, e.g., angiotensin-converting enzyme inhibitors (ACE inhibitors)/angiotensin receptor blockers, angiotensin-receptor-neprilysin inhibitors, beta blockers, and aldosterone antagonists in accordance with current heart failure guidelines.

### Statistics

The SPSS 26 software was used for statistical analysis. Numerical values are expressed as mean ± standard deviation (SD). Median (interquartile range, IQR) was used for continuous variables with a non‐normal distribution, and as frequency (%) for categorical variables. The Kolmogorov–Smirnov test was used to assess normal distributions. All variables in Table 1 were evaluated for the primary study end point in a univariate Cox proportional hazard model. All variables with a significant association were entered in a multivariate Cox model to identify independent predictors of VT/VF. Results are present as hazard risk. A *p*-value < 0.05 was considered significant. All probability values reported are 2-sided.

**Table 1:** Baseline characteristics of the cohort

Variables	*n* = 1675
Demographics	
Male, *n* (%)	1326/1675 (79.2)
Age, mean ± SD	59.3 ± 14.8
Comorbidities	
Coronary artery disease, *n* (%)	466/967 (48.2)
Myocardial infarction, *n* (%)	348/1078 (32.3)
CABG, *n* (%)	103/1156 (8.9)
Chronic obstructive pulmonary disease, *n* (%)	103/886 (11.6)
Chronic kidney disease/Dialysis, *n* (%)	130/967 (13.4)
Atrial fibrillation or atrial flutter, *n* (%)	391/1398 (28)
TIA/stroke, *n* (%)	82/887 (9.2)
Diabetes mellitus, *n* (%)	276/1074 (25.7)
Smoker, *n* (%)	440/1074 (41)
Hypertension, *n* (%)	618/1263 (48.9)
Hyperlipidemia, *n* (%)	520/967 (53.8)
Overweight, *n* (%)	639/1117 (57.2)
BMI kg/m^2^, mean ± SD	27.9 ± 6.6
Family history of cardiovascular disease, *n* (%)	126/889 (14.2)
Hospital side parameters	
Cardiogenic shock at diagnosis, *n* (%)	156/886 (17.6)
Pulmonary edema, *n* (%)	128/886 (14.4)
Days of hospitalization, mean ± SD	13.6 ± 11.1
Drug treatment	
ACE Inhibitors, *n* (%)	811/1157 (70.1)
ARNI, *n* (%)	211/1159 (18.2)
Aldosterone antagonist, *n* (%)	754/1158 (65.1)
ß blocker, *n* (%)	1440/1550 (92.9)
Amiodarone, *n* (%)	173/1559 (11.1)
Clinic treatment results	
Magnetic resonance imaging, *n* (%)	559/1094 (51.1)
Late gandolinium enhancement, *n* (%)	269/416 (64.7)
LVEF and NYHA classification	
LVEF at index, mean ± SD	30 ± 11.9
LVEF short-term, mean ± SD	37.8 ± 12.1
LVEF long-term, mean ± SD	42.6 ± 12.5
EF Improvement > 35%, *n* (%)	1009/1488 (67.8)
Improved LVEF	
No improvement, *n* (%)	436/1515 (28.8)
Improvement in 3 Month, *n* (%)	729/1515 (48.1)
Improvement in 6–12 months, *n* (%)	156/1515 (10.3)
Declined LVEF, *n* (%)	154/1515 (10.2)
Unknown, *n* (%)	40/1515 (2.6)
NYHA at index, *n* (%)	
1	169/1157 (14.6)
2	313/1157 (27.1)
3	484/1157 (41.8)
4	191/1157 (16.5)
NYHA short-term, *n* (%)	
1	281/865 (32.5)
2	397/865 (45.9)
3	172/865 (19.9)
4	15/865 (1.7)
NYHA long-term, *n* (%)	
1	97/225 (43.1)
2	79/225 (35.1)
3	34/225 (15.1)
4	15/225 (6.7)
BNP at baseline (pg/ml), mean ± SD	5554.2 ± 16,582.9
BNP short-term (pg/ml), mean ± SD	2404.8 ± 7052.8
BNP long-term (pg/ml), mean ± SD	1854.6 ± 6372.0

*SD* standard deviation, *CABG* coronary artery bypass grafting, *TIA* transient ischaemic attack, *LVEF* left ventricular ejection fraction, *BMI* body mass index, *COPD* chronic obstructive pulmonary disease, *ACE* angiotensin-converting enzyme, *ARNI* angiotensin-converting enzyme inhibitors, *BNP* brain natriuretic peptide

## Results

### Characteristics of the WCD cohort

**Fig. 1:**
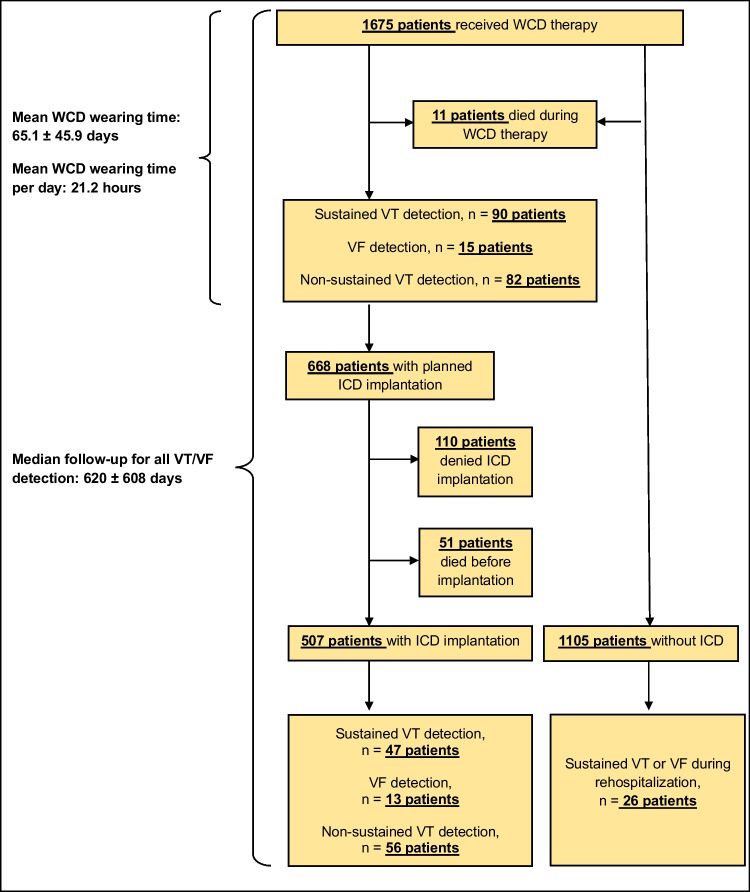
Flowchart presenting the ventricular tachycardia and ventricular fibrillation rates of patients with and without ICD

The cohort of the multicenter registry consisted of a total of 1675 patients, the majority of whom were male (79.2%). The mean age was 59.3 ± 14.8 years. Four hundred forty days (IQR 120–893) was the median follow-up time for the entire cohort (Fig. 1).

The most common indication for WCD therapy was ICM (37%), followed closely by NICM (36%). Other indications in descending frequency were cardiac implantable electronic device (CIED) explantation (9%), myocarditis (8%), unspecified indications (8%), and congenital heart disease and ion channel diseases (2%).

Nearly half of the patient cohort suffered from coronary artery disease and one in three had suffered at least one acute coronary syndrome. A quarter of the patients had diabetes mellitus and about half had arterial hypertension and dyslipidemia. At the time the indication for WCD therapy was established, 17.6% presented with cardiogenic shock and 14.4% with pulmonary edema.

**Fig. 2:**
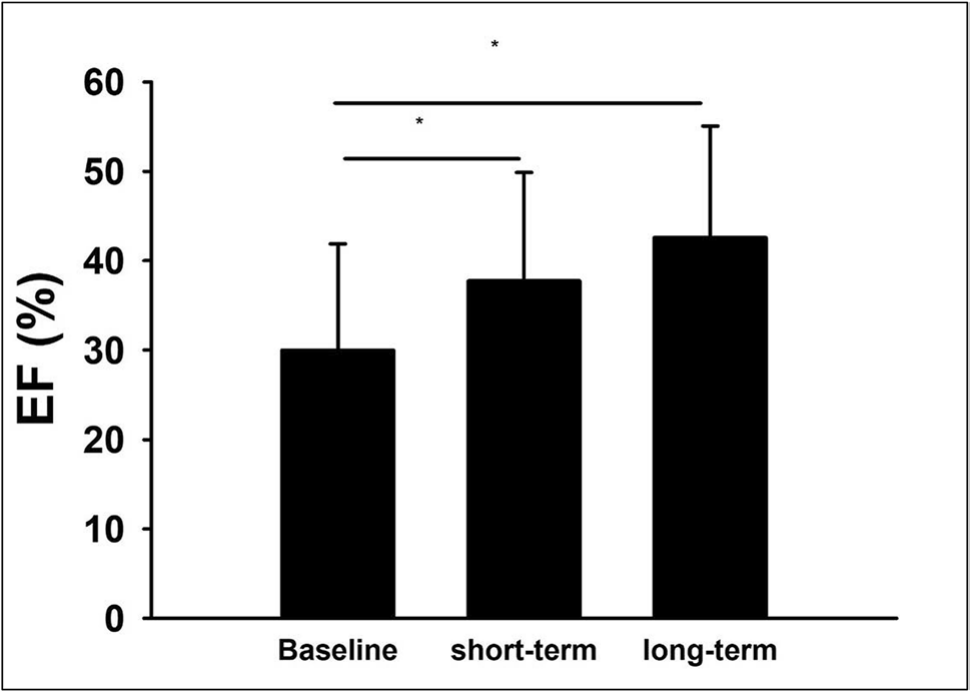
Left ventricular ejection fraction at baseline and during follow-up. *p < 0.05

**Fig. 3:**
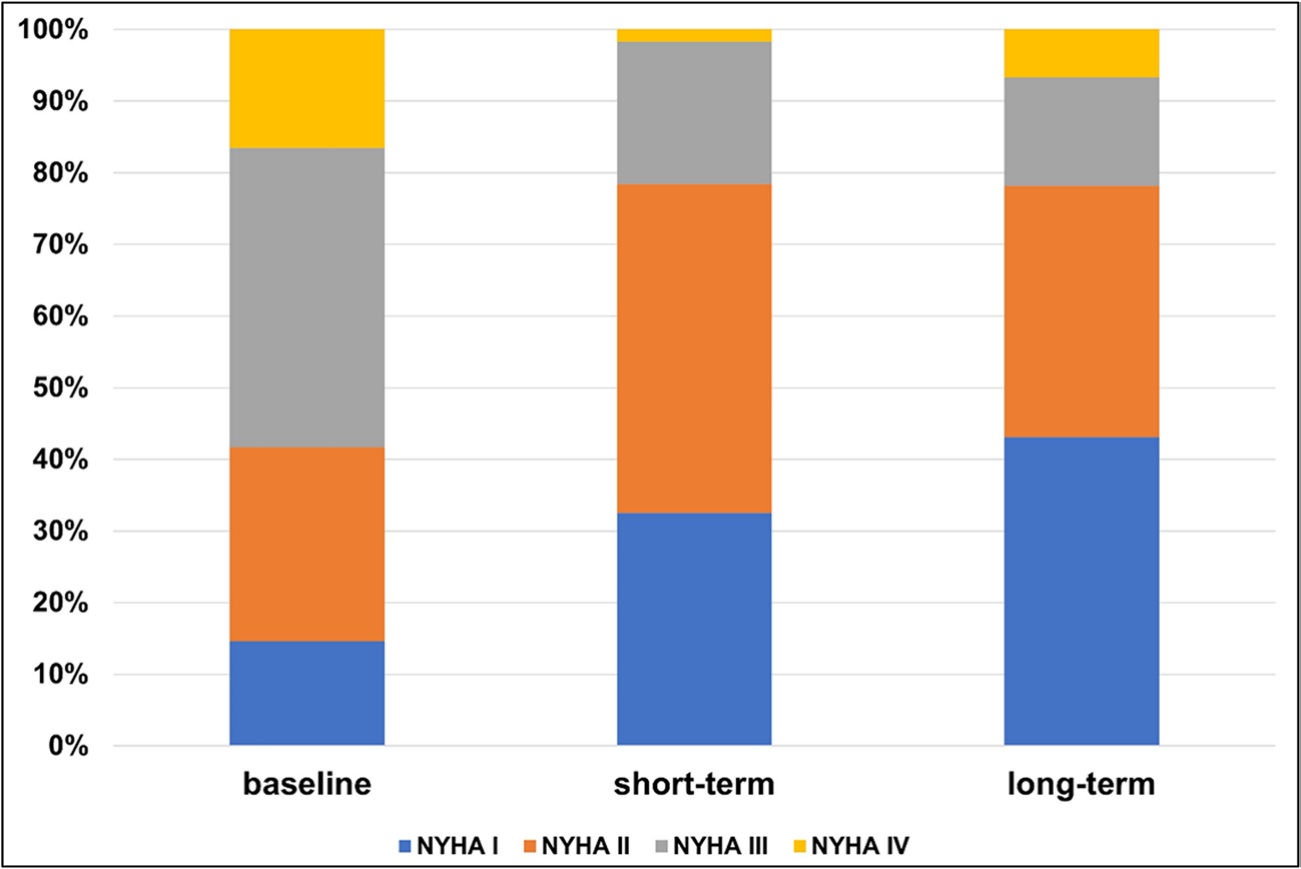
NYHA classification levels at baseline and during follow-up. NYHA I: baseline and short-term: *p* < 0.001; baseline and long-term: *p* < 0.001. NYHA II: baseline and short-term: *p* < 0.001; baseline and long-term: *p* < 0.001. NYHA III: baseline and short-term: *p* < 0.001; baseline and long-term: *p* < 0.001. NYHA IV: baseline and short-term: *p* < 0.001; baseline and long-term: *p* = 0.001

**Fig. 4:**
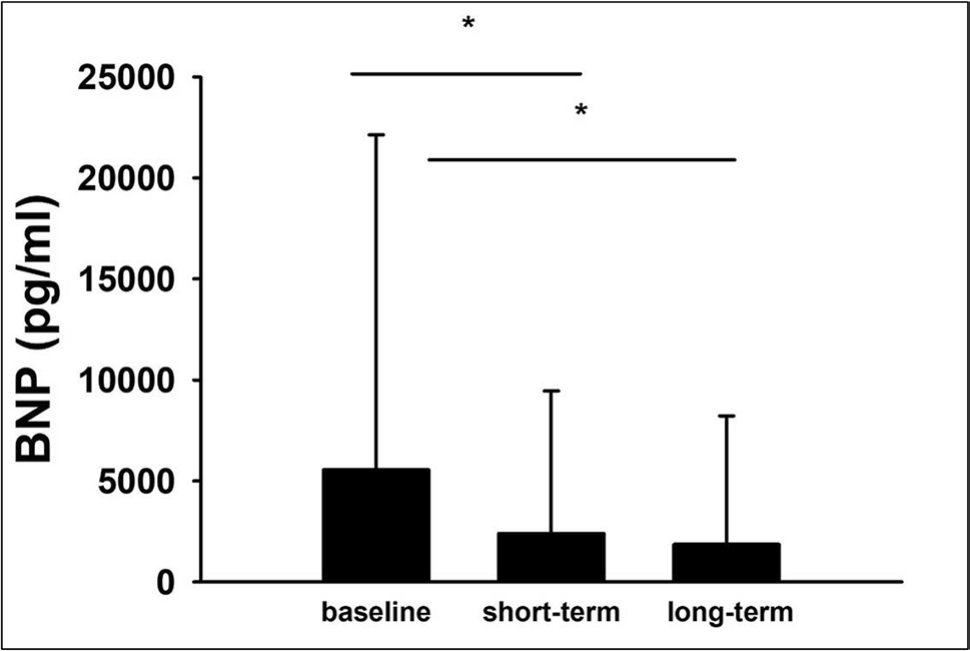
BNP levels at baseline and during follow-up. *p < 0.05

Mean baseline LVEF was 30% and increased to 42.6% at follow-up after 6–12 months (Fig. 2). Of the patients who showed LVEF improvement at follow-up, the largest proportion experienced this improvement in the first three months, while 10.2% had further LVEF improvement after 6–12 months. At the time of indication for WCD therapy, most patients had a more severe NYHA stage (NYHA I, 14.6%; NYHA II, 27.1%; NYHA III, 41.8%; NYHA IV, 16.5%). During follow-up, however, the distribution shifted toward lower NYHA stages (NYHA I, 43.1%; NYHA II, 35.1%; NYHA III, 15.1%; NYHA IV, 6.7%) (Fig. 3). Mean BNP values decreased significantly from the index event to follow-up (from 5554.2 ± 16,582.9 pg/ml at baseline to 1854.6 ± 6372.0 pg/ml in the long-term) (Fig. 4).

Heart failure medication consisted of a beta blocker in 92.9% of patients, an ACE inhibitor in 70.1%, and an aldosterone antagonist in 65.1%. 18.2% received an angiotensin-neprilysin inhibitor. Anti-arrhythmic drug therapy, e.g., amiodarone, was prescribed in 11.1% of the patients (Table 1).

At the time of WCD therapy indication, nearly 25% of patients presented with left bundle branch block on electrocardiogram (ECG) and 7.6% with right bundle branch block.

### WCD data

The mean wearing time of the WCD was 65.1 ± 45.9 days and the mean wearing time per day was 21.2 h. About four out of five patients were compliant and wore the WCD at least 20 h per day. One in four patients wore the WCD for more than three months.

Sustained VT was detected by the WCD in 5.4% of all patients, whereas VF was detected in 0.9% and non-sustained VT in 4.9% (Fig. 1).

Forty-four patients (2.6%) received an appropriate WCD shock; four patients (0.2%) received an inappropriate shock (Supplemental Table 1).

In 5.9% of patients, atrial fibrillation and/or atrial flutter were detected by the device during the WCD wearing period. It was not distinguished whether it was pre-existing or newly onset atrial fibrillation and/or atrial flutter.

The most common reasons for discontinuation of WCD therapy were improvement in LVEF (40.2%), followed by (planned) definitive ICD placement or left ventricular assist device placement (34.2%). Eleven patients died during WCD therapy (1%) (Table 2).

**Table 2:** Wearable cardioverter defibrillator data

Variables	*n* = 1675
Recorded WCD data	
Wear days, mean ± SD	65.1 ± 45.9
Average wear hours, mean ± SD	21.2 ± 4.4
More than 90 wear days, *n* (%)	428/1666 (25.7)
Compliance (> 20 h per day of wear time), *n* (%)	1321/1675 (78.9)
Arrhythmic episodes during WCD	
Ventricular tachycardia, *n* (%)	90/1669 (5.4)
Ventricular fibrillation, *n* (%)	15/1667 (0.9)
Non-sustained ventricular tachycardia, *n* (%)	82/1668 (4.9)
WCD shocks	
Appropriate, *n* (%)	44/1670 (2.6)
Inappropriate, *n* (%)	4/1670 (0.2)
Inhibition of shocks, *n* (%)	521/1393 (37.4)
Recorded atrial fibrillation or atrial flutter by WCD, *n* (%)	73/1241 (5.9)
Recorded AV-block or asystole by WCD, *n* (%)	12/1116 (1.1)
Reason for stopping WCD	
Improved LVEF, *n* (%)	436/1085 (40.2)
Implantation/planed device implantation/ LVAD, *n* (%)	371/1085 (34.2)
Incompliance, *n* (%)	70/1085 (6.5)
Death, *n* (%)	11/1085 (1)
Normal three-month follow-up after MI (%)	21/1085 (1.9)
Unknown, *n* (%)	132/1085 (12.2)
Decision pending, *n* (%)	22/1085 (2)
No further complications with normal LVEF, *n* (%)	19/1085 (1.8)

*SD* standard deviation, *LVAD* left ventricular assist device, *MI* myocardial infarction

### ICD implantation and follow-up

Five hundred seven patients underwent ICD implantation. One hundred ten patients denied indicated permanent ICD-therapy. Fifty-one patients died before ICD implantation could be performed (Fig. 1).

Of the patients who received ICD therapy, sustained VT was detected by the ICD in 47 patients (9.3%), VF in 13 patients (2.6%), and non-sustained VT in 56 patients (11.0%) (Fig. 1).

Four hundred two patients were re-hospitalized during the follow-up, including 142 patients for cardiovascular causes. A total of 104 patients died during the total follow-up. Seven patients suffered arrhythmic death and 29 non-arrhythmic death. In the majority (*n* = 36) of patients, the cause of death was unknown (Table 3).

**Table 3:** Cardiac implantable electronic device implantation and follow-up

Variables	*n* = 1675
Cardiac implantable electronic devices	
Device implantation, *n* (%)	507/1612 (31.5)
Planned implantation, *n* (%)	94/1612 (2.7)
Died before implantation, *n* (%)	51/94 (54.3)
Patient denied, *n* (%)	110/1612 (6.8)
Reported shocks, *n* (%)	59/395 (14.9)
Arrhythmic episodes post-CIED	
Ventricular tachycardia, *n* (%)	47/359 (13.1)
Ventricular fibrillation, *n* (%)	13/359 (3.6)
Others, *n* (%)	21/359 (5.8)
Unknown, *n* (%)	18/359 (5)
Non-sustained ventricular tachycardia, *n* (%)	56/305 (18.4)
Follow-up data	
Death during follow-up, *n* (%)	104/1666 (6.2)
Arrhythmic death, *n* (%)	7/59 (11.9)
No arrhythmic death, *n* (%)	29/59 (49.2)
Indetermined death, *n* (%)	19/59 (32.2)
Rehospitalization, *n* (%)	402/1032 (39)
Cardiovascular cause of rehospitalization, *n* (%)	142/258 (55)
Stroke, *n* (%)	7/258 (2.7)
Ventricular tachycardia/ventricular fibrillation, *n* (%)	26/258 (10.1)
Congestive heart failure, *n* (%)	26/258 (10.1)
Atrial fibrillation, *n* (%)	39/258 (15.1)
Any other cause, *n* (%)	97/258 (37.6)

*CIED* cardiac implantable electronic device

### Predictors of ventricular tachyarrhythmias

Tables 4 and 5 present stepwise Cox regression analyses for identification of predictors of sustained VT/VF. NICM (hazard ratio (HR) 0.5, 95% confidence interval (CI) 0.3–0.6, *p* < 0.001), and medication with ACE inhibitors (HR 0.7, 95% CI 0.6–1.0, *p* = 0.027) and aldosterone antagonists (HR 0.7, 95% CI 0.5–0.9, *p* = 0.005) were associated with a significantly lower risk of VT/VF during follow-up.

**Table 4:** Multivariable analysis for the composite endpoint all sustained ventricular tachycardia and ventricular fibrillation

	Univariable analysis			Multivariable analysis		
	HR	95%CI	*p*-value	HR	95%CI	*p*-value
Male	1.0	0.8–1.3	0.963			
Age	1.0	1.0–1.0	0.733			
Myocarditis	1.6	1.1–2.2	**0.006**	1.3	0.9–1.8	0.222
Ischemic cardiomyopathy	1.0	0.8–1.3	0.914			
Non-ischemic cardiomyopathy	0.4	0.3–0.5	** < 0.001**	0.5	0.3–0.6	** < 0.001**
Atrial fibrillation/Atrial flutter during WCD therapy	1.6	1.1–2.4	**0.017**	1.5	1.0–2.3	0.069
LVEF improvement in the first 3 months	0.6	0.5–0.8	** < 0.001**	0.8	0.6–1.1	0.170
Diabetes mellitus	1.0	0.7–1.3	0.767			
Smoker	0.9	0.7–1.1	0.402			
Hypertension	1.0	0.8–1.3	0.774			
Medication: aspirin	1.2	1.0–1.5	0.110			
Medication: ACE inhibitors	0.6	0.5–0.8	** < 0.001**	0.7	0.6–1.0	**0.027**
Medication: aldosterone antagonists	0.5	0.4–0.7	** < 0.001**	0.7	0.5–0.9	**0.005**
Medication: beta blockers	0.6	0.4–0.8	** < 0.001**	1.1	0.7–1.6	0.778
QRS at baseline	1.0	1.0–1.0	0.233			
QTc at baseline	1.0	1.0–1.0	0.416			

*ACE inhibitors* angiotensin-converting enzyme inhibitors, *CI* confidence interval, *HR* hazard ratio, *LVEF* left ventricular ejection fraction, *WCD* wearable cardioverter defibrillator

**Table 5:** Multivariable analysis for the composite endpoint sustained ventricular tachycardias/ventricular fibrillation detected by wearable cardioverter defibrillator

	Univariable analysis			Multivariable analysis		
	HR	95%CI	*p*-value	HR	95%CI	*p*-value
Male	1.2	0.7–1.9	0.562			
Age	1.0	1.0–1.0	0.485			
Myocarditis	0.8	0.4–1.7	0.629			
Explantation of CIED	2.8	1.7–4.7	** < 0.001**	2.3	1.0–5.1	**0.041**
Ischemic cardiomyopathy	1.2	0.8–1.8	0.359			
Non-ischemic cardiomyopathy	0.4	0.3–0.7	**0.001**	0.3	0.1–0.7	**0.009**
Atrial fibrillation/atrial flutter during WCD therapy	3.3	1.5–7.1	**0.002**	3.3	1.5–7.4	**0.004**
LVEF ≤ 35% at baseline	0.6	0.4–1.0	**0.038**	2.2	0.7–6.7	0.171
Diabetes mellitus	1.6	0.9–3.0	0.126			
Smoker	1.1	0.6–2.0	0.736			
Hypertension	1.1	0.6–1.9	0.707			
Medication: aspirin	1.2	0.7–2.2	0.572			
Medication: ACE inhibitors	0.9	0.5–1.6	0.716			
Medication: aldosterone antagonists	0.8	0.4–1.4	0.366			
Medication: beta blockers	0.6	0.3–1.2	0.126			
BNP levels elevated at baseline	1.0	0.4–2.5	0.981			
QRS at baseline	1.0	1.0–1.0	0.056			
QTc at baseline	1.0	1.0–1.0	0.433			

*ACE inhibitors* angiotensin-converting enzyme inhibitors, *CI* confidence interval, *CIED* cardiac implantable electronic device, *HR* hazard ratio, *LVEF* left ventricular ejection fraction, *WCD* wearable cardioverter defibrillator

Focusing exclusively on VT/VF detected by WCD, NICM was also associated with a significantly lower risk (HR 0.3, 95% CI 0.1–0.7, *p* = 0.009), while atrial fibrillation and/or atrial flutter during WCD therapy (HR 3.3, 95% CI 1.5–7.4, *p* = 0.004), as well as explantation of a cardiac-implantable electronic device (HR 2.3, 95% CI 1.0–5.1, *p* = 0.041), were associated with a significantly higher risk of VT/VF (Graphical abstract).

## Discussion

We present baseline clinical characteristics as well as outcome data of 1675 patients with an estimated high risk of SCD treated with a WCD with a median follow-up of 440 days, including identification of predictors of sustained VT/VF.

The main findings of the study are the following: (1) a total of 187 episodes of ventricular tachyarrhythmia were detected by the WCD (including 90 episodes of sustained VT, 15 of VF, and 82 of non-sustained VT); (2) additional 116 episodes of ventricular tachyarrhythmia were detected in patients who received ICD implantation (including 47 episodes of sustained VT, 13 of VF, and 56 of non-sustained VT); and (3) NICM and medication with aldosterone antagonists and ACE inhibitors were associated with a significantly lower risk of VT/VF.

The WCD may be considered in selected patients at high risk for SCD when implantation of a conventional ICD is temporarily not indicated (e.g., low LVEF after acute coronary syndrome or myocarditis until LV function improves, after ICD explantation because of bacteremia). The American Heart Association, American College of Cardiology, Heart Rhythm Society, and European Society of Cardiology classify the use of WCD in these selected patients as class II level C evidence [1, 16]. The rate of appropriate WCD shocks in this study was 2.6%. Results of the European Heart Rhythm Association survey revealed that many hospitals hesitate to use WCDs due to a questionable cost–benefit ratio [17]. Current data remain unclear, especially as previous studies on cost-utility analyses focused primarily on patients with ICD explantation [18]. Here it was shown that WCD therapy is likely to be more cost-effective in protecting patients from SCA after infected ICD removal during the wait for ICD reimplantation than keeping patients in hospital or discharging them home or to a care facility, but rates of effective WCD shocks are higher in this patient cohort [18].

### Non-ischemic cardiomyopathy is associated with a lower risk for ventricular arrhythmia

The DANISH trial evaluated primary prophylactic ICD implantation in NICM patients. The results demonstrated that ICD implantation in these patients did not significantly reduce the risk of death from any cause. However, the rate of SCD was significantly lower and a large proportion of the study population received a cardiac resynchronization therapy device [19]. Similarly, the DEFINITE trial compared ICD + OMT versus OMT alone in patients with NICM. Again, ICD implantation was associated with a significantly lower risk of arrhythmogenic death with comparable all-cause mortality in both groups [20].

In NICM, there is a significant risk of ventricular tachyarrhythmias and cardiac death in the first few weeks during initiation and optimization of heart failure therapy [21, 22]. WCD therapy may be an effective temporary prophylaxis to prevent SCD in patients with newly diagnosed NICM and significantly impaired LVEF until a decision about definitive ICD placement can be made [21, 22].

In the WEARIT-II study including 2000 patients, NICM patients had a significantly lower risk of sustained ventricular arrhythmias during 3 months of WCD use than patients with ICM or patients with congenital/inherited heart disease [23]. One-year follow-up of this study demonstrated that all-cause mortality was lowest in the NICM group. Interestingly, patients with ventricular tachyarrhythmia detected by WCD had a significantly increased 1-year mortality (10% vs 3%, *p* = 0.042) [24]. Our study is consistent with these findings as we identified NICM as an independent predictor of lower rates of ventricular arrhythmia episodes compared to other aetiologies.

A subgroup analysis of the WEARIT II trial examined the clinical course of patients with WCD use ≤ 90 days compared with WCD use > 90 days. In NICM patients, however, rates of sustained ventricular arrhythmias were comparable with WCD use > 90 vs ≤ 90 days (13.4 vs 13.7 events per 100 patient-years, *p* = 0.314). Thus, this suggests that patients with prolonged WCD use of > 90 days remain at risk for ventricular arrhythmias [25]. The PROLONG study investigated the use of WCD to reduce early ICD implantation in patients with newly diagnosed heart failure. The study showed that intensified optimization of heart failure therapy, supported by prolonged WCD wear time, may help prevent the need for early ICD implantation, as a relative proportion of patients showed LVEF improvement only after 3 months, particularly in patients with NICM [14]. Improving LVEF in the first three months was associated with a significantly lower risk of VT/VF in our study.

In summary, evidence regarding the outcome of NICM patients with WCD is still limited. On the one hand, this study demonstrated that NICM as an indication for WCD therapy is associated with significantly lower rates VT/VF than other indications, which indicates that these patients may be prevented from ICD implantation by prolonged WCD use and improving LVEF during the period, but on the other hand, there is also evidence that NICM patients may remain at risk for VT/VF even after 90 days of WCD use. Thus, it should be considered that under prolonged WCD protection, it may be beneficial to continue to increase the dose of heart failure medication because LVEF may further improve.

### ACE inhibitors, aldosterone antagonists, and the risk of ventricular arrhythmia

In our predictor analysis, we demonstrate that the use of ACE inhibitors and aldosterone antagonists were associated with a significantly lower risk of VT/VF occurrence in the total follow-up.

ACE inhibitors have consistently shown a reduction in overall mortality in clinical trials and systematic reviews involving patients with heart failure [26]. Surprisingly, ACE inhibitors did not exhibit a significant decrease in SCD, with moderate-quality evidence to support this finding [27, 28].

On the other hand, two clinical trials investigating the use of aldosterone antagonists in heart failure with reduced ejection fraction, namely RALES and EMPHASIS-HF, demonstrated a substantial 25–30% reduction in the risk of all-cause mortality and cardiovascular death [29, 30]. In the EPHESUS trial, eplerenone also exhibited significant reductions in SCD (4.9% vs. 6.1%), as well as in total mortality, particularly during the early post-infarction period (first 30 days), when patients face the highest risk of SCD [31]. Furthermore, a meta-analysis, combining data from individual patient levels across the three placebo-controlled randomized trials (RALES, EMPHASIS-HF, and EPHESUS) involving 11,032 patients, indicated that aldosterone antagonist treatment was associated with a 23% reduction in the risk of SCD among heart failure patients with LV systolic dysfunction when compared to placebo [32].

The important significance of maximizing the use of OMT in heart failure patients with reduced LVEF is once again emphasized. Importantly, in our predictor analysis, the use of ACE inhibitors and aldosterone antagonists was associated with a significantly lower risk for the occurrence of VT/VF throughout the follow-up period, but not during the WCD wearing period. Thus, it can be concluded that short-term use of OMT may not be as effective as long-term use, i.e., these drugs have a protective effect after a minimum of 3 months.

OMT for heart failure patients has changed significantly in recent years. Newer agents such as angiotensin-neprilysin inhibitors and sodium-glucose-transporter-2 inhibitors have significantly improved treatment [33–35]. The inclusion period of the present WCD registry covers almost 10 years, which might explain that the number of patients taking these two medications remained relatively small and that, as a result, an effect on the occurrence of VT/VF could not be observed in this analysis.

However, the use of baseline medical therapy as a potential risk factor for ventricular arrhythmic events may be limited. An association without causality may have been identified as treatment with aldosterone antagonists and angiotensin-neprilysin inhibitors was likely prescribed to patients with more favorable baseline characteristics who are more likely to tolerate OMT and experience fewer adverse arrhythmic and clinical events.

### The impact of atrial fibrillation and atrial flutter on ventricular tachyarrhythmia

In our multivariable analysis, atrial fibrillation/atrial flutter during WCD therapy was an independent predictor of VT/VF. This finding could be explained by the fact that the risk for VT/VF and SCD is significantly increased in patients with atrial fibrillation [36]. Okin et al. demonstrated that new-onset atrial fibrillation was associated with a more than fourfold increased risk of SCD [37]. Overall, however, the relationship between atrial fibrillation and SCD is difficult to address because atrial fibrillation is also a risk factor for other cardiac conditions, such as coronary artery disease and heart failure. These, in turn, are the two most significant causes of ventricular tachyarrhythmias and SCD [38]. Several studies have shown that the risk of VT/VF and SCD is significantly increased in patients with atrial fibrillation and that heart failure is an additional, strong risk factor [39–41]. This may explain the association between atrial fibrillation and SCD [41]. Atrial fibrillation could therefore be an indicator of worsening heart failure or lead to acute decompensation of preexisting heart failure, resulting in a higher rate of VT/VF.

Most patients in the present WCD registry had ICM or NICM with severely impaired LVEF, particularly during the WCD wearing period. Therefore, it is not surprising that atrial fibrillation/ atrial flutter was a significant risk factor for VT/VF detected by WCD in our analysis. However, this is the first study including patients at high risk of SCD that could support this association.

## Limitations

Despite the advantages of the present registry and the predictor analysis that emerged from it, some limitations should be highlighted: first, the retrospective nature of the data collection and analysis; second, the heterogeneity of the data because patients were enrolled in several centers in two European countries. Consequently, there is no complete follow-up in some patients, particularly in patients without ICD implantation, i.e., the cause of death is unfortunately unknown in some cases, and it remained undetermined whether the death was arrhythmic or non-arrhythmic. Third, OMT for heart failure has changed and improved significantly over the past decade. Patients enrolled at the beginning of the registry study may have received different medical therapy than patients enrolled more recently. Finally, we did not evaluate other diagnostic methods such as magnetic resonance imaging to identify patients at high risk for VT/VF and SCD.

## Conclusion

The present predictor analysis of the WCD registry revealed that patients suffering from NICM and receiving OMT for heart failure, especially ACE inhibitors and aldosterone antagonists, had a significantly lower risk of sustained VT/VF. On the other hand, the presence of atrial fibrillation and atrial flutter increased the likelihood of detecting VT/VF. The results emphasize the importance of heart failure OMT, during the long-term, not only for LVEF improvement but also for preventing the occurrence of life-threatening VT/VF in patients in whom ICD implantation is being evaluated. The predictors may be useful to identify patients who, on the one hand, may benefit from WCD therapy because they are at increased risk for VT/VF (e.g., patients with atrial fibrillation) and, on the other hand, are more likely to be at lower risk but in whom WCD therapy protects them from SCD in the acute phase during the period of OMT optimization and LVEF improvement (e.g., patients with NICM). Particularly in the latter, ICD implantation might thus be avoided.

## Supplementary Information

Below is the link to the electronic supplementary material.

Supplementary file1 (DOCX 25 kb)

## Data Availability

The data underlying this article will be shared on reasonable request to the corresponding author.

## Abbreviations

ACE inhibitors: Angiotensin-converting enzyme inhibitors
CIED: Cardiac implantable electronic device
ECG: Electrocardiogram
ICD: Implantable cardioverter defibrillator
ICM: Ischemic cardiomyopathy
LVEF: Left ventricular ejection fraction
NICM: Non-ischemic cardiomyopathy
OMT: Optimal medical treatment
SCD: Sudden cardiac death
VF: Ventricular fibrillation
VT: Ventricular tachycardia
WCD: Wearable cardioverter defibrillator
