# Supplementary material for: Predictors of ventricular tachyarrhythmia in patients with a wearable cardioverter defibrillator: an international multicenter registry
Source: J Interv Card Electrophysiol. 2024 Jul 10;67(8):1917–28. doi: 10.1007/s10840-024-01869-w (PMC11606999; doi:10.1007/s10840-024-01869-w)
Supplement: Supplementary file 1 — Supplementary file1 (DOCX 25 kb) [file 10840_2024_1869_MOESM1_ESM.docx]

**44 patients with an appropriate WCD shock:**

| **Patient** | **Gender** | **Age** | **WCD indication** | **LVEF at baseline (%)** | **Arrhythmic episode at WCD shock** | **ICD implant** | **Appropriate ICD shock** | **Death during FU** | **Medication** |
| --- | --- | --- | --- | --- | --- | --- | --- | --- | --- |
| 1 | M | 66 | ICM | 35 | VT | Yes | Yes | No | Aspirin, Prasugrel, statin, ACE inhibitor or ARB, betablocker, Amiodarone, Diuretics |
| 2 | F | 31 | Myocarditis | 53 | VT | Yes | No | Unknown | Betablocker |
| 3 | M | 48 | ICD-Explant | 30 | VT | Yes | No | No | ACE inhibitor or ARB, Aldosterone antagonist, betablocker, diuretics |
| 4 | M | 53 | ICM | 25 | VF | Yes | No | No | Aspirin, Prasugrel, statin, ACE inhibitor or ARB, Aldosterone antagonist, betablocker, diuretics |
| 5 | M | 64 | ICM | 35 | VT | Yes | Yes | No | Rivaroxaban, statin, ACE inhibitor or ARB, betablocker, diuretics |
| 6 | M | 75 | ICM | 20 | VT | Yes | No | No | Aspirin, Clopidogrel, statin, ACE inhibitor or ARB, Aldosterone antagonist, betablocker, diuretics |
| 7 | F | 75 | ICM | 30 | VT | Yes | Unknown | No | Unknown |
| 8 | F | 88 | ICM | 25 | VT | Yes | Unknown | Unknown | Unknown |
| 9 | M | 64 | ICD-Explant | 25 | VT | Yes | No | Yes | ACE inhibitor or ARB, Amiodarone, diuretics, Procoralan |
| 10 | M | 71 | ICD-Explant | 27 | VT | Yes | Yes | No | Clopidogrel, statin, betablocker, Amiodarone, diuretics, Procorolan |
| 11 | M | 60 | NICM | 13 | VF | Yes | No | No | Statin, ACE inhibitor or ARB, Aldosterone antagonist, betablocker, Amiodarone, diuretics, Procoralan |
| 12 | M | 64 | ICD-Explant | 40 | VF | Patient denied |  | No | Betablocker |
| 13 | F | 84 | ICM | 35 | VT | No |  | No | Betablocker |
| 14 | M | 62 | ICD-Explant | 20 | VT | Yes | Unknown | No | Betablocker |
| 15 | F | 55 | Indeterminate | 55 | VF | Patient denied |  | No | Betablocker |
| 16 | M | 84 | ICM | 27 | VT | No |  | No | Betablocker |
| 17 | M | 25 | Congenital heart disease | 60 | VT | Patient denied |  | No | Betablocker |
| 18 | M | 78 | NICM | 30 | VT | Planed |  | Unknown | Betablocker |
| 19 | M | 64 | ICD-Explant | 40 | VT | No |  | No | Betablocker |
| 20 | M | 67 | ICM | 35 | VT | Yes | Unknown | Unknown | Betablocker, Amiodarone |
| 21 | F | 31 | Myocarditis | 53 | VT | Planed |  | Unknown | Betablocker |
| 22 | M | 48 | NICM | 30 | VT | Patient denied |  | No | Betablocker |
| 23 | M | 54 | ICM | 25 | VT | Yes | Unknown | No | Betablocker |
| 24 | M | 64 | ICM | 35 | VT | Patient denied |  | No | Betablocker |
| 25 | M | 75 | NICM and ICM | 20 | VT | Patient denied |  | No | Betablocker |
| 26 | F | 74 | NICM | 16 | VF | Yes | No | No | ACE inhibitor or ARB, Aldosterone antagonist, betablocker, diuretics |
| 27 | F | 61 | ICD-Explant | 25 | VF | Planed |  | No | Aspirin, Clopidogrel, Phenprocoumon, statin, Amiodarone |
| 28 | M | 67 | Indeterminate | 35 | VT | Yes | Yes | No | Phenprocoumon, ACE inhibitor or ARB, Aldosterone antagonist, betablocker, Amiodarone |
| 29 | M | 77 | ICM | 29 | VT | No |  | No | Aspirin, Clopidogrel, statin, ACE inhibitor or ARB, Aldosterone antagonist, betablocker, diuretics |
| 30 | M | 82 | ICM | 20.3 | VT | Yes | Yes | No | Aspirin, Clopidogrel, statin, ARNI, betablocker, diuretics |
| 31 | F | 44 | ICD-Explant | 75.4 | VF | Yes | No | No | Apixaban, diuretics |
| 32 | M | 51 | ICM | 33 | VT | Yes | No | No | Aspirin, Prasugrel, statin, ARNI, Aldosterone antagonist, betablocker, Amiodarone, diuretics |
| 33 | M | 77 | ICM | 26 | VF | Unknown |  | Unknown | Aspirin, Clopidogrel, statin, ACE inhibitor or ARB, Aldosterone antagonist, diuretics |
| 34 | F | 25 | Indeterminate | 35 | VT | Yes | Yes | No | ACE inhibitor or ARB, Aldosterone antagonist, betablocker, Amiodarone, |
| 35 | M | 78 | ICM | 14.8 | VT | Unknown |  | Unknown | Clopidogrel, Phenprocoumon, statin, ACE inhibitor or ARB, betablocker, Amiodarone, diuretics, Digitalis |
| 36 | M | 58 | ICM | 25.7 | VF | Yes | No | No | Clopidogrel, statin, ARNI, Aldosterone antagonist, betablocker, diuretics, Digitalis |
| 37 | M | 41 | NICM | 29.7 | VT | No |  | No | Aspirin, Clopidogrel, statin, ACE inhibitor or ARB, Aldosterone antagonist, betablocker, diuretics |
| 38 | F | 51 | Myocarditis | 30 | VT | Yes | Yes | No | Rivaroxaban, ACE inhibitor or ARB, Aldosterone antagonist, beta blocker, Amiodarone, diuretics |
| 39 | M | 81 | ICM | Unknown | VT and VF | Yes | No | No | Statin, ACE inhibitor or ARB, betablocker, Amiodarone, diuretics |
| 40 | F | 58 | ICM | 26.6 | VT and VF | Yes | Unknown | No | Apixaban, Clopidogrel, statin, ACE inhibitor or ARB, Aldosterone antagonist, betablocker, Amiodarone, diuretics |
| 41 | M | 66 | ICM | 35 | VT and VF | Yes | Yes | Yes | Aspirin, Prasugrel, statin, ACE inhibitor or ARB, betablocker, diuretics |
| 42 | M | 55 | ICM | 49 | VT | Yes | Unknown | No | Aspirin, Ticagrelor, statin, betablocker |
| 43 | F | 82 | Indeterminate | 45 | VT | Yes | Yes | No | ACE inhibitor or ARB, Amiodarone |
| 44 | M | 72 | ICD-Explant | 39 | VT | Yes | Unknown | No | ACE inhibitor or ARB, Aldosterone antagonist, betablocker |

ACE, angiotensin-converting-enzyme; ARB, aldosterone receptor blocker; ARNI, angiotensin receptor neprilysin inhibitor; F, female; ICD, implantable cardioverter defibrillator; ICM, ischemic cardiomyopathy; M, male, NICM, non-ischemic cardiomyopathy; VF, ventricular fibrillation; VT, ventricular tachycardia
